# Supplementary material for: A novel technique for olfactory bulb measurements
Source: PLoS One. 2020 Dec 16;15(12):e0243941. doi: 10.1371/journal.pone.0243941 (PMC7743925; doi:10.1371/journal.pone.0243941)
Supplement: Attachment — Submitted filename: Response to reviewers.docx [file pone.0243941.s002.docx]

Dear Reviewers and Editors,

- Thank you very much for your time and comment on the manuscript.

Reviewer #1: In this manuscript, Joshi et al demonstrate a technique for delineating the olfactory bulb volume in MRI scans of the human brain. Whereas the standard technique takes a well-trained observer 10 minutes of tracing, the new technique requires less expertise and time. The demonstration that the two techniques give equivalent results is promising. However, given that the techniques were only applied by the study's co-authors, I wonder if this equivalence can be put to a more stringent test. Would the two techniques give equivalent results if applied by less expert observers? Since one of the claimed advantages of the box-frame technique is that it requires less expertise, it would make sense to determine whether box-frame gives equivalent performance for less-expert observers. I suggest that the authors ask other observers, preferably not co-authors of the study, to apply the two techniques. The value of the box-frame technique will be more compelling if its advantages generalize to non-authors.

In response to the valuable comments raised by the reviewers; two non- experts belonging to completely different background with no imaging experience measured the bulb volumes using box- frame approach in total study population. Further, reliability was tested between measurements done by experts and non- experts. Cronbach alpha values shows that the technique is worth relying on and therefore supports its universal usability.

I would like to mention the changes made to the manuscript.

1) In introduction, page 3, line number 4:

^(3)^ checking usability of the new technique by experts and non- experts.

2) In methods, page 4, line number 21:

The idea for proposing the BF approach was also its usability by non-experts in neuroimaging. Accordingly, we checked its performance by non- expert observers who belonged to a different background with no imaging experience. They were well explained how the technique works and were asked to do the measurements in all of the subject population. Following the same rules, when the difference exceeded 10%, a third non-expert observer calculated the volumes again.

3) In table 1, added mean ± Stdev of the measurements done by non- experts:

| **OB results using the Box- Frame method (non-expert)** |  |
| --- | --- |
| Volume of right OB (Observer 1) (in mm^3^) | 33.65 ± 17.78 |
| Volume of right OB (Observer 2) (in mm^3^) | 39.24 ± 22.10 |
| Volume of left OB (Observer 1) (in mm^3^) | 42.12 ± 24.46 |
| Volume of left OB (Observer 2) (in mm^3^) | 42.71 ± 27.23 |

4) In results, page 7, line number 14:

For BF approach, inter- observer reliability was checked for measurements done by experts and non- experts. The Cronbach’s alpha (α) for right OB was 0.82 and 0.83 for left OB. The results advantages its usability by non- experts or less trained as well.

5) In discussion on page 7, line 25 added:

and when used by experts and non- experts.

- In response to the point raised by the editor, changes were made in Acknowledgement section.

On page 8, line number 30:

Many thanks to three non-expert observers for their time, efforts and cooperation.
